# Computational drug repositioning using meta-path-based semantic network analysis

**DOI:** 10.1186/s12918-018-0658-7

**Published:** 2018-12-31

**Authors:** Zhen Tian, Zhixia Teng, Shuang Cheng, Maozu Guo

**Affiliations:** 1 School of Information Engineering, Zhengzhou University, Zhengzhou, 450001 People’s Republic of China; 2 School of information and computer engineering, Northeast Forestry, Harbin, 150001 People’s Republic of China; 3 Institute of Materials, China Academy of Engineering Physics, Jiang You, 621907 Sichuan China; 4 School of Electrical and Information Engineering, Beijing University of Civil Engineering and Architecture, Beijing, 100044 People’s Republic of China; 5 Beijing Key Laboratory of Intelligent Processing for Building Big Data, Beijing, 100044 China

**Keywords:** Semantic network analysis, Drug repositioning, Meta-path-based, HeteSim, HSDD

## Abstract

**Background:**

Drug repositioning is a promising and efficient way to discover new indications for existing drugs, which holds the great potential for precision medicine in the post-genomic era. Many network-based approaches have been proposed for drug repositioning based on similarity networks, which integrate multiple sources of drugs and diseases. However, these methods may simply view nodes as the same-typed and neglect the semantic meanings of different meta-paths in the heterogeneous network. Therefore, it is urgent to develop a rational method to infer new indications for approved drugs.

**Results:**

In this study, we proposed a novel methodology named HeteSim_DrugDisease (HSDD) for the prediction of drug repositioning. Firstly, we build the drug-drug similarity network and disease-disease similarity network by integrating the information of drugs and diseases. Secondly, a drug-disease heterogeneous network is constructed, which combines the drug similarity network, disease similarity network as well as the known drug-disease association network. Finally, HSDD predicts novel drug-disease associations based on the HeteSim scores of different meta-paths. The experimental results show that HSDD performs significantly better than the existing state-of-the-art approaches. HSDD achieves an AUC score of 0.8994 in the leave-one-out cross validation experiment. Moreover, case studies for selected drugs further illustrate the practical usefulness of HSDD.

**Conclusions:**

HSDD can be an effective and feasible way to infer the associations between drugs and diseases using on meta-path-based semantic network analysis.

## Background

Over the past decades, de novo drug development is expensive, time-consuming and limited to a relatively small number of targets [1–3]. By conservative estimate, the cost for developing a new drug is about $1.8 billion dollars, and the developing time is about 15 years [4]. To overcome these problems, researchers and pharmaceutical enterprises have begun to pay their attentions to finding new medical indications from those approved drugs [5]. Drug repositioning (or drug repurposing), which can identify new indications of existing drugs, is able to offer a promising alternative to minimize costs and risks for drug discovery [6, 7]. At the same time, several successfully repositioned drugs have shown that such drug repositioning is an effective way (one example is Minoxidil) [8, 9]. What’s more, since elucidating the molecular basis of disease on a personalized level has become an attainable goal, drug repositioning will play a key role in drug discovery and precision medicine paradigm [10, 11].

With the generation of large-scale genomic, transcriptomic and proteomic data, it has become a feasible way to predict new drug-disease associations based on computational models [12]. These methods can be mainly divided into three catalogues: machine learning-based approaches, network-based approaches and text mining and semantic inference approaches [13]. Here, we will present a brief review for each category. A detailed review is beyond the scope of this paper and has already been presented by Li [13] and Shahreza [14].

Machine learning-based models make the best use of biological data in publicly databases for the prediction of novel associations for drugs and diseases [15]. Firstly, drugs will be represented by features vectors, which are derived from their properties, such as drug fingerprint, chemical structures and side effects, while diseases are characterized by phenotype data [16]. Then machine learning-based models are trained based on various features of drugs and diseases. Lastly, we can predict associations of drugs and diseases based on these learning-based models.

Gottlieb et al [5] firstly proposed a novel method called PREDICT for the large-scale prediction of drug indications. The proposed method employed multiple drug-drug and disease-disease similarity measures to construct a logistic regression classifier for drug repositioning. Menden [17] mainly made use of both genomic features of the cell lines and chemical properties of considered drugs, which aims to build a feed-forward perceptron neural network model for the sake of solving the drug repositioning problem. Inspired by Menden, Napolitano et al [18] put forward a drug-centered computational approach, which utilized the integrated drug chemical structures similarity, drug molecular target similarity and drug-gene expression similarity to complete the prediction. Besides, Zhang [19], Yang [20], Wu [21] and Liang [22] also put forward their respectively machine learning models to infer drug-disease associations.

At the same time, network-based methods are widely used strategy for computational drug repositioning [23–25]. While traditional study mostly focuses on exploring the shared characteristics among drug compounds such as chemical structures [26] and side-effects [9], recent network-based approaches [27] take pharmacological, genetic and clinical data into account to explore the relationships between drugs and diseases from network point view. The assumption of network-based methods is that similar drugs are normally associated with similar diseases and vice versa. Therefore, measuring the similarity between disease phenotypes is essential for drug repositioning [28]. One of the most commonly used rules is guilt-by-association (GBA) in association relationship prediction [29].

Cheng [30] developed three supervised inference methods which are called drug-based similarity inference (DBSI), target-based similarity inference (TBSI) and network-based inference (NBI) respectively, to predict both drug-target interactions and drug-disease associations. These methods made use of the structural similarity, target-target genomic sequence similarity and drug-target topology network similarity. Wu et al [31] built a weighted disease and drug heterogeneous network with the disease-gene and drug-target relationships from the KEGG database. They clustered the weighted network to identify modules and then assembled all possible drug-disease pairs based on the processed modules. Huang [32] adopted the idea of data fusion and integrated three different networks of drug, genomic and disease phenotype with available experimental data and knowledge. The proposed method inferred drug-disease associations by means of network propagation approach. More recently, Luo [33] proposed a novel computational method named MBiRW to identify potential novel indications for a given drug. MBiRW mainly developed comprehensive similarity measures for drugs and diseases to infer the drug-disease associations. Experimental results on various datasets demonstrated that the proposed approach has a reliable prediction performance. Besides, other methods [1, 8, 12, 34] are also employed to predict novel drug and target associations based on biological networks and achieved great successes.

Except for machine learning-based and network-based approaches, text mining and semantic inference methods are also effective in predicting drug-disease associations. Especially with the rapid development in text mining research, it is a possible manner to detect novel indications for existing drugs [35, 36]. Exploring the associations of drugs and diseases from biomedical literature, MEDLINE and knowledge databases about genes, has become a meaningful way. Similar to machine learning-based and network-based methods, these methods [37, 38] can be an effective way in addressing drug repositioning problems.

Although network-based methods have been used in drug repositioning successfully, most of these approaches simply view objects (nodes) in drug-disease heterogeneous networks as the same type. What’s more, these methods do not consider the different semantic meaning of meta-paths, which is crucial for the prediction performance of network-based methods. For example, Luo [33] built a heterogeneous network by integrating similarities between drugs and disease as well as the known drug-disease association network. A novel Bi-Random walk is developed to identify new indications for existing drugs. However, the algorithm treated all the edges in the heterogeneous network equally. Indeed, edges in drug similarity network and disease similarity network represent the similarity relationship of drugs and diseases, while the edges in the drug-disease association network represent the association relationships. The values of edges in the similarity network range from 0 to 1, while values of edges in the drug-disease association network is 0 or 1. This negligence may lead to deviations in predicting results.

Machine learning-based models need to find the information of drugs such as fingerprint, chemical structure and so on. Then drugs can be represented by comprehensive vectors respectively. In this way, we can solve drug-repositioning problems by utilizing all kinds of effective machine learning models such as deep learning. However, machine learning-based models needs to build highly credible negative datasets firstly, which is quite difficult for current data. Network-based methods measure the similarities between drugs and diseases to construct comprehensive similarity networks. Similarity measurement models are employed to settle drug-repositioning problems. While these methods don’t utilize the negative samples like machine learning-based methods, they have to mining potential associations in depth. Text mining and semantic inference methods mainly explore the associations of drugs and diseases from biomedical literature. In other words, the associations obtained by these methods are all supported by literature, which is alternative to solve drug-repositioning problem. Therefore, these three methods can make up for each other.

HeteSim [39] is a path-based measure which can accurately measure the relatedness of nodes with the same or different types in a heterogeneous network. This method can effectively capture semantics of meta-paths, which is crucial for measuring the relevance of nodes in heterogeneous networks [40–43].

**Fig. 1:**
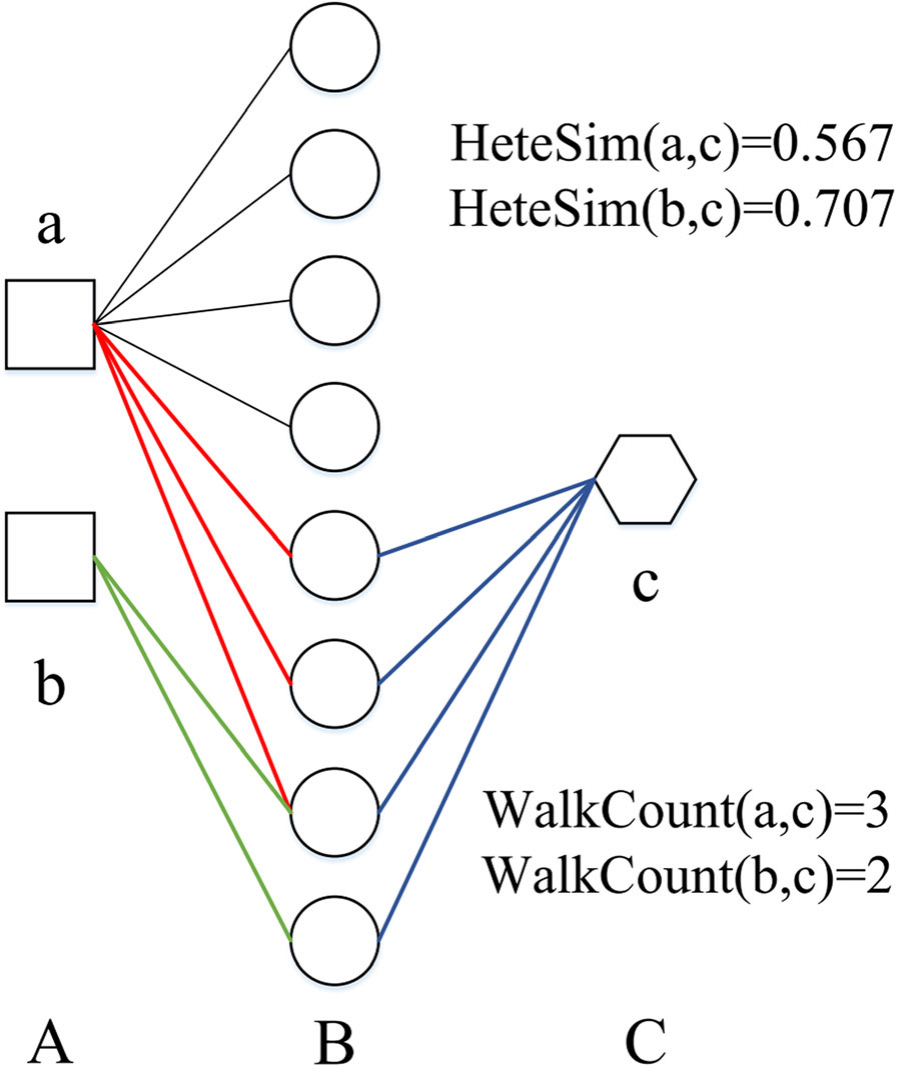
Example of the heterogeneous network for comparing walk count and HeteSim measure. Circles, squares and sexangle denotes three types of object, which are (**a**, **b** and **c**) respectively

Methods Katz [44] and CATAPULT [45] only use walk count to measure the similarity between objects, which is shown in Fig. 1. The walk-count between *a* and *c* is larger than *b* and *c*, which indicates that *a* is closer to *c* than *b*. The association strength between *a* and *c*, *b* and *c* is 3 and 2 based on walk count, respectively. However, we find that the connections starting from node *a* possess less meaning than the connections starting from node *b*. Intuitively, the connectivity between *b* and *c* should more intense than *a* and *c*, which is in accordance with the results of HeteSim. The association strength between *a* and *c*, *b* and *c* is 0.567 and 0.707, respectively. Therefore, the similarity calculated by the HeteSim measure seems to be a more reasonable result, which can effectively obtain the semantic meaning of different meta-paths.

In this paper, we proposed a novel method called HeteSim_DrugDisease (HSDD) based on HeteSim scores to measure the associations of drugs and diseases. We first construct a heterogeneous network consisting of the drug-drug similarity network, the drug-disease association network and a disease-disease similarity network. Then, we employ the HeteSim approach to measure the relatedness scores for drug-disease pairs considering the semantic meaning of meat-paths. In the end, we utilize HSDD to predict drug-disease associations. The detail description of HSDD is presented in Methods Section.

## Methods

### Datasets

To construct a drug-disease heterogeneous network, we downloaded information of drugs and diseases from different data sources. The data mainly contains phenotype similarity network, drug similarity network and drug-disease associations. Next, we will briefly introduce the data used in our experiment. The experimental data used is shown in Table 1.

**Table 1:** The summary table for data used in this article

	drug similarity network	disease similarity network	drug-disease association network
size	663 × 663	5080×5080	540×306
edge value	(0,1)	(0,1)	0,1

#### Disease similarity network

We derived the disease similarity network from MimMiner [46], which is measured based on disease phenotypes. Each disease has one or more phenotype types in the OMIM database [47]. According to the MimMiner database description, the phenotype similarities in the network are measured based on text mining approaches. The similarity values have been normalized to the range [0,1]. Furthermore, we adopt a logistic transformation process to modify the phenotypic similarities, which have been proposed by Vanunu [48]. The definition of the logistic function is

$$ L(x)=\frac{1}{1+\exp \left( cx+d\right)} $$

where *x* denotes the similarity value between phenotypes in MimMiner database, *c*and *d*are the parameters. In this study, we set *c*and *d*as − 15 and log(9999) respectively. From the equation above, we can find that small similarity values will be transformed to be close zero and large similarity values will be enlarged.

#### Drug-disease association network

The drug-disease association network used in this study was obtained from Gottlieb et al [5]. In this gold standard dataset, there are totally 1933 known drug drug-disease associations involving 593 drugs registered in DrugBank database [49] and 313 diseases listed in Online Mendelian Inheritance in Man (OMIM) [47]. In this study, there are totally 1776 associations related 540 drugs and 306 diseases.

#### Drug similarity network

The drug similarity network was obtained from the supplementary material of the paper [33]. The authors made the best use of the chemical structures of drugs, similarity correlation analysis and sharing information between drugs to construct a comprehensive drug similarity network, which has totally 663 drugs in this original drug similarity network. The similarity values of drugs range from 0 to 1.

### Construction of the drug-disease heterogeneous network

In drug similarity network, let *DR* = {*dr*_1_, *dr*_2_, …, *dr*_*m*_} denotes the set of *m* drugs. The similarity between *dr*_*i*_and *dr*_*j*_ can be denoted by *sim*(*dr*_*i*_, *dr*_*j*_). Similar to drugs, let *DI* = {*di*_1_, *di*_2_, …, *di*_*n*_}denotes the set of *n* diseases in the disease similarity network. The comprehensive similarity value can be represented by *sim*(*di*_*i*_, *di*_*j*_).

**Fig. 2:**
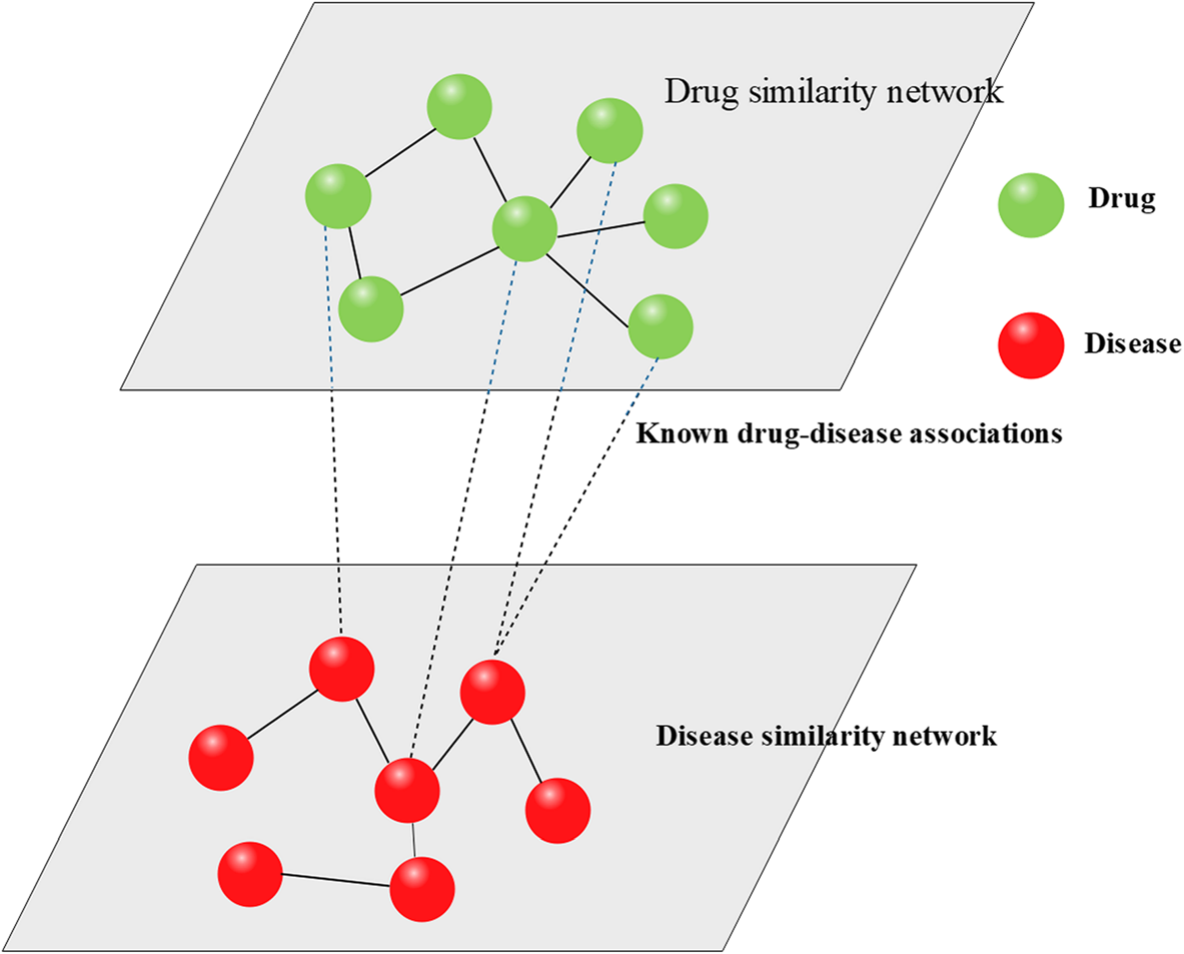
The drug-disease heterogeneous network model. The green nodes denote drugs and red nodes denote diseases. Dash lines represent the known drug-disease associations while the solid lines represent the similarities of drugs or diseases

The drug-disease association network can be represented by a bipartite graph *G*(*V*, *E*), where *V*(*G*) = {*DR*, *DI*} and *E*(*G*) is the edge set which contains all the similarities of drugs and diseases and associations between drugs and diseases. If *dr*_*i*_ is associated with *di*_*j*_ is 1, the weight of edge between them is 1, otherwise, the weight of edge between them is 0. Then we can construct a drug-disease heterogeneous network, which is presented in Fig.2.

Suppose the matrices *D*, *Q* and *P* denote the matrices for drug similarity network, drug-disease association network and disease similarity network respectively, the drug -disease heterogeneous network can be expressed as

$$ H=\left[\begin{array}{cc}D& Q\\ {}{Q}^T& P\end{array}\right] $$

where *Q*^*T*^denotes the transpose of matrix *Q*.

### HeteSim description

Given a relevance path called *S* = (*A*, *R*), which is denoted by ${A}_1\overset{R_1}{\to }{A}_2\overset{R_2}{\to}\cdots \overset{R_l}{\to }A{}_{l+1}$. The composite relation between *A*_1_ and *A*_*l* + 1_is defined as *R* = *R*_1_ ∘ *R*_2_ ∘ ⋯ ∘ *R*_*l*_. *A*_*i*_ refers to one of type nodes in the heterogeneous network and *R*_*i*_ refers to the relationship between *A*_*i*_ and*A*_*i* + 1_. For simplicity, we can also use the type name denoting the relevance path such as *P* = (*A*_1_*A*_2_⋯*A*_*l* + 1_), when there is only one relation between pairs.

The HeteSim score between two objects *s*(*s* ∈ *R*_1_. *A*_1_) and *t*(*t* ∈ *R*_*l*_. *A*_*l* + 1_) based on the relevance path *R* = *R*_1_ ∘ *R*_2_ ∘ *R*_3_ ∘ ⋯ ∘ *R*_*l*_, which is expressed as

1 $$ {\displaystyle \begin{array}{l} HeteSim\left(s,t\left|P\right.\right)= HeteSim\left(s,t\left|R\right.\right)= HeteSim\left(s,t\left|{R}_1\circ \right.{R}_2\circ {R}_3\circ \cdots \circ {R}_l\right)\\ {}\kern6em =\frac{1}{\left|O\left(s\left|{R}_1\right.\right)\right|\left|I\left(t\left|{R}_l\right.\right)\right|}\sum \limits_{i=1}^{\left|O\left(s\left|{R}_1\right.\right)\right|}\sum \limits_{j=1}^{\left|I\left(t\left|{R}_l\right.\right)\right|} HeteSim\Big(\left({O}_i\left(s\left|{R}_1\right.\right)\right),{I}_j\left(t\left|{R}_l\right.\right)\left|{R}_2\circ {R}_3\circ \cdots \circ {R}_{l-1}\Big)\right.\kern1.25em \end{array}} $$

where *O*(*s*|*R*_1_) is the out-neighbors of *s* based on relation *R*_1_ and *I*(*t*|*R*_*l*_) is the in-neighbors of *t* based on relation *R*_*l*_. From the eq. (1), we can find that computation of *HeteSim*(*s*, *t*|*P*) needs to iterate over all pairs (*O*_*i*_(*s*|*R*_1_), *I*_*j*_(*t*|*R*_*l*_)) of (*s*, *t*) along the path and sum up the relatedness of these pairs [39]. Then, we normalize it by the total number of out-neighbors of *s* and in-neighbors of *t*. That means the relevance between *s* and *t* is the average relevance between out-neighbors *s* and in neighbors of *t*.

Specially, the HeteSim score between two same-typed objects *s* and *t* based on the self-relation *I* is

$$ HeteSim\left(s,t\left|I\right.\right)=\delta \left(s,t\right) $$

where *δ*(*s*, *t*) = 1 if *s* sand *t* are same-typed objects, or else*δ*(*s*, *t*) = 0. Obviously, this is not appropriate for our study. Therefore, Yang [50] re-defined HeteSim score on self-relation as the similarity or association strength if *s* and *t* is associated, otherwise as 0.

The meta-paths in heterogeneous networks have semantic meanings, which make the relatedness of two same-typed objects depending on the given relevance path. Therefore, HeteSim has the ability to measure the similarity of two nodes in a heterogeneous accurately.

### Calculation of HeteSim scores

**Definition 1**. *Transition probability matrix. SupposeA and B are two object types in a heterogeneous network,* (*W*_*AB*_)_*n* × *m*_*is the adjacent matrix between typeA and B. The transition probability matrix of A* → *B can be expressed as*

$$ {U}_{AB}\left(i,j\right)=\frac{W_{AB}\left(i,j\right)}{\sum_{k=1}^m{W}_{AB}\left(i,k\right)} $$

$$ {V}_{AB}\left(i,j\right)=\frac{W_{AB}\left(i,j\right)}{\sum_{k=1}^m{W}_{AB}\left(k,j\right)} $$

Matrix *U*_*AB*_ is the normalized results of matrix *W*_*AB*_ along the row vector and *V*_*AB*_ is the normalized results of matrix *W*_*AB*_along the column vector. It is easy to prove that *U*_*AB*_ is equal to ${V}_{BA}^{\prime }$.

**Definition 2**. *Reachable probability matrix. In a heterogeneous network, given an arbitrary relevance path P* = *A*_1_*A*_2_⋯*A*_*l* + 1_*and two objects s* ∈ *A*_1_*andt* ∈ *A*_*l* + 1_*, a reachable probability matrix for path P* = *A*_1_*A*_2_⋯*A*_*l* + 1_*is defined as,*

$$ {R}_P={U}_{A_1{A}_2}{U}_{A_2{A}_3}\cdots {U}_{A_l{A}_{l+1}} $$

Objects *s* and *t* will meet at the middle type node when *s* follows along the path and *t* goes against the path. When the length of path *P* is even, *s* and *t* will meet at the middle of node*A*_(*l*/2) + 1_. The path *P* = (*A*_1_*A*_2_⋯*A*_*l* + 1_) can be divided into two equal-length parts as *P* = (*P*_*L*_*P*_*R*_),where*P*_*L*_ = (*A*_1_*A*_2_⋯*A*_*mid* − 1_*A*_*mid*_) and *P*_*R*_ = (*A*_*mid*_*A*_*mid* + 1_⋯*A*_*l* + 1_). Here *mid* = (*l*/2) + 1. When the length of path *P* is odd, *s* and *t* will not be meet at the same node. In this study, we adopt a compromised method which is proposed by Zeng [42].

Finally, the HeteSim score between *s*(*s* ∈ *R*_1_. *A*_1_) and *t*(*t* ∈ *R*_*l*_. *A*_*l* + 1_) based on the path *P* is calculated as follows:

2 $$ {\displaystyle \begin{array}{l} HeteSim\left(s,t\left|P\right.\right)= HeterSim\left({A}_1,{A}_{l+1}\left|{P}_L,{P}_R\right.\right)\\ {}={U}_{A_1{A}_2}{U}_{A_2{A}_3}\cdots {U}_{A_{mid-1}M}{V}_{MA_{mid+1}}{V}_{A_{mid+1}{A}_{mid+2}}\cdots {V}_{A_l{A}_{l+1}}\\ {}={U}_{A_1{A}_2}{U}_{A_2{A}_3}\cdots {U}_{A_{mid-1}M}{U}_{MA_{mid+1}}^{\prime }{U}_{A_{mid+1}{A}_{mid+2}}^{\prime}\cdots {U}_{A_l{A}_{l+1}}^{\prime}\\ {}=\left({U}_{A_1{A}_2}{U}_{A_2{A}_3}\cdots {U}_{A_{mid-1}M}\right){\left({U}_{A_l{A}_{l+1}}\cdots {U}_{A_{mid+1}{A}_{mid+2}}{U}_{MA_{mid+1}}\right)}^{\prime}\\ {}=\left({PM}_{P_L}\right){\left({PM}_{P_R^{-1}}\right)}^{\prime}\kern20.75em \end{array}} $$

where ${PM}_{P_L}={U}_{A_1{A}_2}{U}_{A_2{A}_3}\cdots {U}_{A_{mid-1}M}$and ${PM}_{P_R^{-1}}={U}_{A_l{A}_{l+1}}\cdots {U}_{A_{mid+1}{A}_{mid+2}}{U}_{MA_{mid+1}}$. In the Eq. (2), the transition probability matrix of *A*_*i*_ → *A*_*j*_, denotes as ${U}_{A_i{A}_j}$, is the row normalized matrix of adjacent matrix${W}_{A_i{A}_j}$, and the transition probability matrix of*A*_*j*_ → *A*_*i*_, ${V}_{A_i{A}_j}$ is the column normalized results of matrix ${W}_{A_i{A}_j}$. The HeteSim score between *s* and *t* along the path *P* can be expressed as

$$ HeteSim\left(s,t\left|P\right.\right)={PM}_{P_L}\left(s,:\right){PM}_{P_R^{-1}}\left(t,:\right) $$

**Definition 3**. *Normalization of HeteSim. The normalized HeteSim score between two objects s and t based on the relevance path P is*

$$ HeteSim\left(s,t\left|P\right.\right)=\frac{PM_{P_L}\left(s,:\right){PM}_{P_R^{-1}}\left(t,:\right)}{\sqrt{\left\Vert {PM}_{P_L}\left(s,:\right)\right\Vert}\sqrt{\left\Vert {PM}_{P_R^{-1}}\left(t,:\right)\right\Vert }} $$

As is stated by Shi [39], the normalized HeteSim is the cosine of probability distributions of source object *s* and target object *t* reaching the middle type object*M*. The HeteSim score ranges from 0 to 1.

### Example for HeteSim

**Fig. 3:**
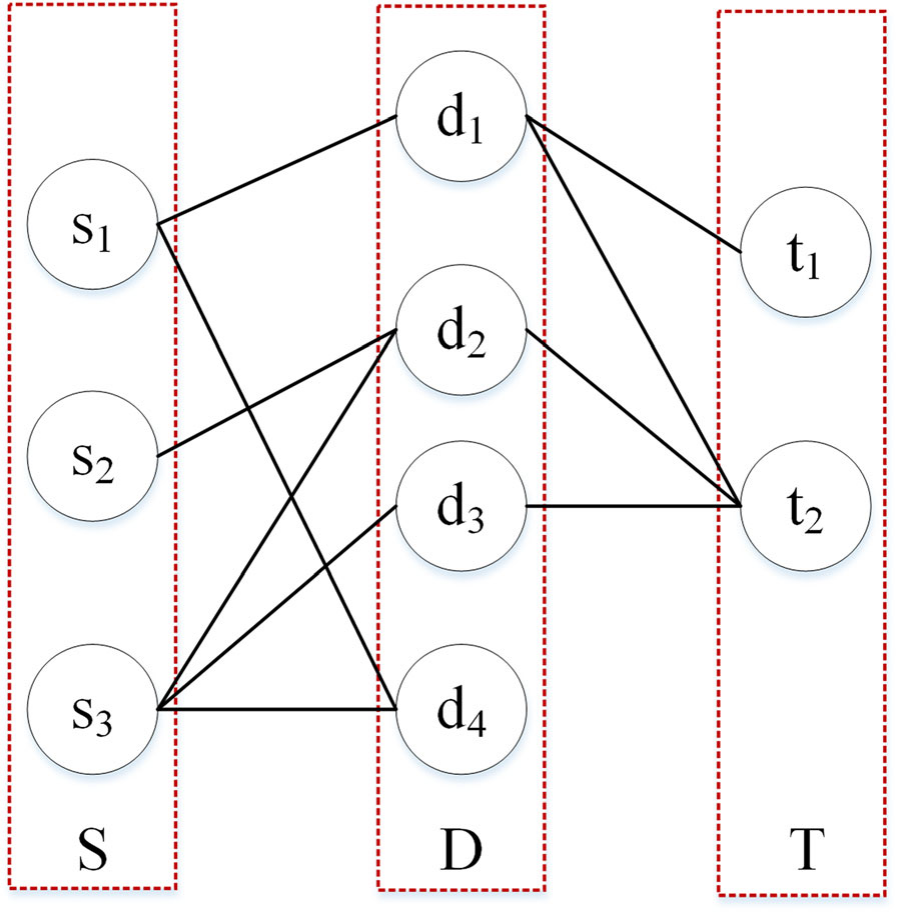
Example for computing HeteSim scores

A heterogeneous network is shown in Fig.3. The heterogeneous network contains three object types. Here, we show the procedure of measuring the HeteSim scores between *s*_1_ and *t*_1_, *t*_2_ under the relevance path *P* = (*SDT*). The path relevance path *P* = (*SDT*) can be divided two parts *P*_*L*_ = (*SD*) and *P*_*R*_ = (*DT*).

The adjacency matrix *W*_*SD*_ and *W*_*TD*_ can be denoted as:

$$ {\displaystyle \begin{array}{l}\kern3em {d}_1\kern0.75em {d}_2\kern0.75em {d}_3\kern0.75em {d}_4\\ {}{W}_{SD}=\begin{array}{c}{s}_1\\ {}{s}_2\\ {}{\mathrm{s}}_3\end{array}\left[\begin{array}{cccc}1& 0& 0& 1\\ {}0& 1& 0& 0\\ {}0& 1& 1& 1\end{array}\right]\end{array}} $$

$$ {\displaystyle \begin{array}{l}\kern3.25em {d}_1\kern0.75em {d}_2\kern0.75em {d}_3\kern0.75em {d}_4\\ {}{W}_{TD}=\begin{array}{c}{t}_1\\ {}{t}_2\end{array}\left[\begin{array}{cccc}1& 0& 0& 0\\ {}1& 1& 1& 0\end{array}\right]\end{array}} $$

Then we normalize the above matrices along the row vector. The transition probability matrix of *S* → *D* and *T* → *D* can be represented as:

$$ {U}_{SD}=\left[\begin{array}{cccc}0.5& 0& 0& 0.5\\ {}0& 1& 0& 0\\ {}0& 0.333& 0.333& 0.333\end{array}\right] $$

$$ {U}_{TD}=\left[\begin{array}{cccc}1& 0& 0& 0\\ {}0.333& 0.333& 0.333& 0\end{array}\right] $$

According to the Eq. (2), the reachable matrices for *P*_*L*_ and *P*_*R*_ are equivalent their transition probability matrices, which is ${V}_{DT}={U}_{TD}^{\prime }$ [39]. Therefore, the HeteSim scores for *s*_1_,*t*_1_ and *s*_1_,*t*_2_based on path *P* can be calculated as:

$$ {\displaystyle \begin{array}{l} HeteSim\left({s}_1,{t}_1\left|P\right.\right)=\frac{PM_{P_L}\left({s}_1,:\right){PM}_{P_R^{-1}}\left({t}_1,:\right)}{\sqrt{\left\Vert {PM}_{P_L}\left({s}_1,:\right)\right\Vert}\sqrt{\left\Vert {PM}_{P_R^{-1}}\left({t}_1,:\right)\right\Vert }}\\ {}\kern6.75em =\frac{U_{SD}\left(1,:\right)\left({V}_{DT}\left(1,:\right)\right)}{{\left\Vert {U}_{SD}\left(1,:\right)\right\Vert}_2\times {\left\Vert \left({V}_{DT}\left(1,:\right)\right)\right\Vert}_2}=\frac{U_{SD}\left(1,:\right){\left({U}_{TD}\left(1,:\right)\right)}^T}{{\left\Vert {U}_{SD}\left(1,:\right)\right\Vert}_2\times {\left\Vert \left({U}_{TD}\left(1,:\right)\right)\right\Vert}_2}=0.707\end{array}} $$

$$ {\displaystyle \begin{array}{l} HeteSim\left({s}_1,{t}_2\left|P\right.\right)=\frac{PM_{P_L}\left({s}_1,:\right){PM}_{P_R^{-1}}\left({t}_2,:\right)}{\sqrt{\left\Vert {PM}_{P_L}\left({s}_1,:\right)\right\Vert}\sqrt{\left\Vert {PM}_{P_R^{-1}}\left({t}_2,:\right)\right\Vert }}\\ {}\kern6.75em =\frac{U_{SD}\left(1,:\right)\left({V}_{DT}\left(2,:\right)\right)}{{\left\Vert {U}_{SD}\left(1,:\right)\right\Vert}_2\times {\left\Vert \left({V}_{DT}\left(2,:\right)\right)\right\Vert}_2}=\frac{U_{SD}\left(1,:\right){\left({U}_{TD}\left(2,:\right)\right)}^T}{{\left\Vert {U}_{SD}\left(1,:\right)\right\Vert}_2\times {\left\Vert \left({U}_{TD}\left(2,:\right)\right)\right\Vert}_2}=0.408\end{array}} $$

### HeteSim_Drug_Disease method

In the drug-disease heterogeneous network used in this study, there are different meta-paths connecting drugs and disease. For example, a drug and a disease phenotype can be connected via “drug-disease phenotype” path and “drug-drug-disease phenotype” path and so on. As we know, these different meta-paths may have different semantic meanings. e.g. “Drug-drug-disease phenotype” path indicates that if a drug is associated with a disease, then other drugs similar to the drug can be regard as the potential drugs associated with the disease. “Drug-disease-disease” path means that if a disease is associated with a drug, the other diseases similar to the disease will be associated with the drug. Next, we will give a systematic introduction to measure the similarity between drugs and diseases connecting by meta-paths.

The proposed method HSDD employs HeteSim to compute the similarity of drugs and diseases in the drug-disease heterogeneous network. Usually, scores of different meta-paths are combined with a constant that dampens contributions from longer path. HeteSim can effectively measure the subtle semantics of meta-paths and we need to combine HeteSim scores of different paths with a constant *β* to dampen the contributions from longer paths. In this paper, the parameter *β* needs to be validated by experiments further.

The similarity of *S*(*s*, *t*) based on HSDD can be expressed as

$$ S\left(s,t\right)=\sum \limits_{l=2}^{\infty }{\beta}^{l-1}\times \sum \limits_{p_i\in {\Psi}_l} HeteSim\left(s,t\left|{p}_i\right.\right) $$

Here *s* and *t* denote one drug and one disease, respectively.Ψ_*l*_denotes the set of paths connecting the drug *s* to the disease phenotype *t* with path length *l*. It is generally believed that a short path may contribute more than a long path. In this study, we only consider the meta-paths with length less than five for HSDD. All the paths that used to measure the association between drugs and diseases are listed in Table 2. There are total 14 paths used for HSDD.

**Table 2:** Paths with length less than five

Path lengths	Pathway scheme	Pathway
2	DrDrDi	drug→drug→disease
	DrDiDi	drug→disease→disease
3	DrDrDrDi	drug→drug→drug→disease
	DrDrDiDi	drug→drug→disease→disease
	DrDiDiDi	drug→disease→disease→disease
	DrDiDrDi	drug→disease→drug→disease
4	DrDrDrDrDi	drug→drug→drug→drug→disease
	DrDrDrDiDi	drug→drug→drug→disease→disease
	DrDrDiDrDi	drug→drug→disease→drug→disease
	DrDrDiDiDi	drug→drug→disease→disease→disease
	DrDiDrDrDi	drug→disease→drug→drug→disease
	DrDiDrDiDi	drug→disease→drug→disease→disease
	DrDiDiDrDi	drug→disease→disease→drug→disease
	DrDiDiDiDi	drug→disease→disease→disease→disease

Given a drug *s* and a disease phenotype *t*, the association strength is measured by

3 $$ {\displaystyle \begin{array}{l}S\left(s,t\right)=\sum \limits_{l=2}^4\left({\beta}^{l-1}\times \sum \limits_{p_i\in {\Psi}_l} HeteSim\left(s,t\left|{p}_i\right.\right)\right)\kern13.5em \\ {}\kern1.75em ={\beta}^{\ast}\left( HeteSim\left(s,t\left| DrDrDi\right.\right)+ HeteSim\Big(s,t\left| DrDiDi\right.\Big)\right)\kern7.25em \\ {}\kern2.75em +{\beta}^{2\ast}\Big( HeteSim\left(s,t\left| DrDrDrDi\right.\right)+ HeteSim\left(s,t\left| DrDrDi Di\right.\right)\kern2.5em \\ {}\kern2.75em + HeteSim\left(s,t\left| DrDiDi Di\right.\right)+ HeteSim\left(s,t\left| DrDiDrDi\right.\right)\Big)+L\\ {}\end{array}} $$

## Results

In this section, we firstly introduce the metrics used to evaluate the performance of various prediction measures. Next, we will perform a comprehensive comparison between HSDD and other representative methods using diseases with known and unknown drugs datasets. After that, we will investigate the effect of parameter *β*and path lengths on HSDD. At last, we conduct case study to verify the effectiveness of HSDD in inferring drug-disease associations.

### Evaluation measures

Firstly, to evaluate the performance of different methods systematically, we conduct a leave-one-out cross validation (LOOCV) experiment. For each drug, at each iteration, one of its drug-disease associations is treat as the test data and all the remaining associations as the training data. After performing prediction, each tested drug ranked together with all other drugs in descending order according to the predicted score. For each specific ranking threshold, if the rank of the tested connection is above the selected threshold, it is regarded as a true positive. The number of true positive over all possible drug-disease relationships is regarded as the true-positive rate corresponding to the specified threshold. On the other hand, if the rank of an unknown connection is above the threshold, it is regarded as a false positive. True-positive rate and false-positive rate are computed with varying ranking thresholds for the sake of constructing the receiver operating characteristic (ROC) curve. Area under curve (AUC) represents the overall performance of the algorithms.

Secondly, it is generally believed that the predicted top-ranked results are also very important and useful in practice. As a result, we compare the performances of all prediction methods in term of the top hundred predicted drugs. The specified top-rank thresholds refers to the thresholds that used to count correctly retrieved drug-disease associations. The specified top-rank thresholds used in this article is discrete, which range from 0 to 1 with scale 0.1. The more true associations in the top portions, the more effective the prediction method is.

Thirdly, meta-paths with different lengths have different contributions to relatedness of drugs and diseases. The parameter *β* in Eq. (3) can dampen the contributions of longer paths. In this study, we will systematically evaluate its effect on HSDD and then tune its best value by cross validations.

Lastly, we conduct a case study experiment, which predicts top-ten related drugs for five common diseases for seeking evidence from biomedical literature to verify the effectiveness of HSDD.

### Comparison with existing methods on disease with known drugs

We compare HSDD with other four representative methods: NBI [30], HGBI [34] and DrugNet [8], MBiRW [33]. As is mentioned in previous section, NBI could prioritize candidate drugs for a given target or prioritize candidate targets for a given drug simultaneously. HGBI predicted new drug-disease relationships in the newly proposed three-layer model by using an information flow-based method. DrugNet is also a network-based drug repositioning method and able to predict both drug-disease and disease-drug prioritization. MBiRW is the state-of-the-art method and can infer potential novel indications for drugs. In this study, we compare HSDD with these four methods, by LOOCV experiment and de novo drug–disease prediction analysis. The parameters in HSDD are that the combined path is with length 2, 3, 4 and *β* equals 0.8.

**Fig.4:**
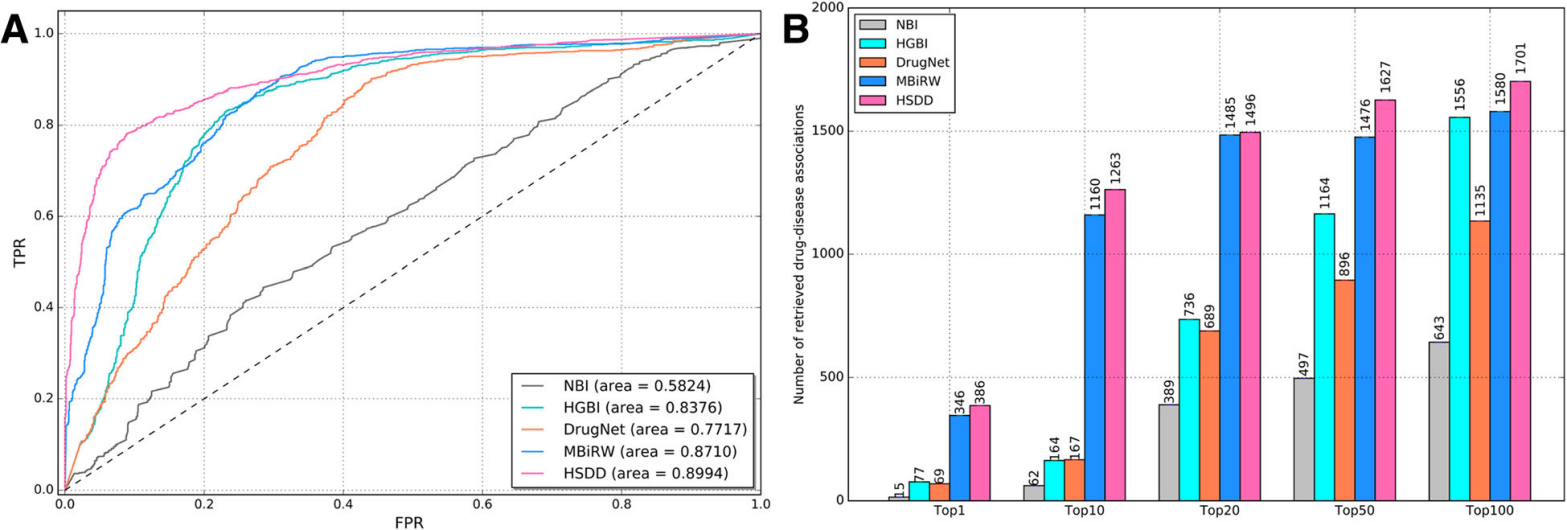
**a** ROC curves for predicting drug–disease associations based on various methods. **b** Number of correctly retrieved known drug–disease associations for various rank thresholds

We conduct the LOOCV experiment for predicting drug-disease associations. In total, there are 1776 drug-disease associations involving 540 drugs and 306 diseases in this experiment. The ROC and AUC values for all methods are presented in Fig. 4a. Method HSDD performs best in the five methods overall. The AUC value for HSDD is 0.8994, while those for methods NBI, HGBI, DrugNet and MBiRW are 0.5824, 0.8376, 0.7717 and 0.8710, respectively.

Moreover, we further investigate the number of correctly retrieved drug-disease associations. A true drug-disease association is considered as correctly retrieved if the predicted ranking of this association is higher than the specified top-rank threshold [33]. The results are shown in Fig. 4b. Method HSDD significantly outperforms the other four compared methods. For HSDD, 386 associations are predicted at the top 1, while the results for NBI, HGBI, DrugNet and MBiRW are 15, 77, 69 and 346, respectively. As for the top 10, top 20, top 50 and top 100 evaluation metric, HSDD also performs best, which is followed by MBiRW. Therefore, HSDD can be more useful in practice than other four approaches.

### De novo drug–disease prediction

**Fig. 5:**
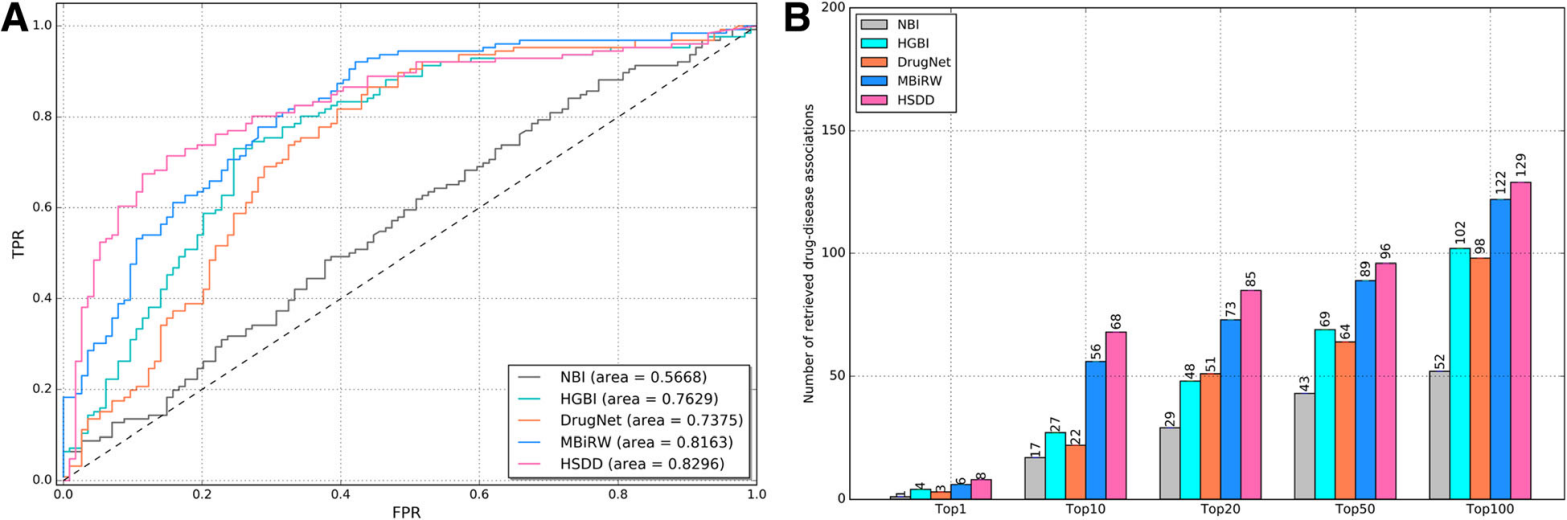
De novo drug–disease prediction. **a** ROC curves for predicting drug–disease associations based on various methods. **b** Number of correctly retrieved known drug–disease associations for various rank thresholds

To evaluate the capability of HSDD in predicting potential indications for new drugs, we conduct the de novo drug-disease prediction test. In this experiment, we select the drugs, which only have one associated disease. There are totally 153 associations in this experiment to evaluate the performance of HSDD by the capability to recover the association. There are totally 153 drugs, 132 diseases, and 153 drug-disease associations used in this experiment. At the same time, we also evaluate the performance of NBI, HGBI, DrugNet and MBiRW. The corresponding results have be presented in Fig. 5.

As is shown in Fig.5a. HSDD achieves an AUC of 0.8296, which outperforms other four methods in the same experimental scenario. The AUC values for NBI, HGBI, DrugNet and MBiRW are 0.5668, 0.7629, 0.7375 and 0.8163, respectively.

Moreover, we also investigate the number of correctly retrieved drug-disease associations. The results are listed in Fig. 5b. From the results, we can find that HSDD also outperforms other four methods. For example, among the 153 known drug-disease associations, HSDD achieves 8 of them at the top 1, while the results for NBI, HGBI, DrugNet and MBiRW is 1, 4, 3, and 6. For top 10, HSDD successfully predicts 68 associations, while the results for NBI, HGBI, DrugNet and MBiRW are 17, 27, 22 and 56, respectively. Overall, all de novo prediction results indicate that HSDD can achieve a superior performance.

### The effect of parameters on HSDD

In this section, we investigate the effect of parameter*β*on HSDD. The parameter *β*dampens the contributions of different length paths. Besides, some research has found that the longer the path length is, the smaller the inhibiting factor is [51]. Therefore, we combine the value of *β* and path lengths as shown in Table 3. The value of *β* ranges from 0.1 to 1.0 with the scale 0.1. We divide the relevance path into two types: combined path and independent path. The combinations between *β* and different path lengths are presented in Table 3. We conducted the LOOCV experiment and calculated the AUC values based on various combinations. The corresponding results are shown in Table 3.

**Table 3:** The AUC values of HSDD under different combinations of parameters

*β*	Path length combinations					
	2	3	4	2,3	3,4	2,3,4
0.1	0.7423	0.7313	0.6613	0.8495	0.8325	0.8525
0.2	0.7439	0.7320	0.6628	0.8506	0.8379	0.8596
0.3	0.7508	0.7387	0.6643	0.8521	0.8396	0.8612
0.4	0.7523	0.7411	0.6667	0.8645	0.8401	0.8659
0.5	0.7611	0.7434	0.6684	0.8702	0.8417	0.8728
0.6	0.7684	0.7460	0.6714	0.8761	0.8436	0.8862
0.7	0.7680	0.7487	0.6731	0.8799	0.8524	0.8934
0.8	0.7689	0.7534	0.6712	0.8831	0.8596	0.8983
0.9	0.7574	0.7423	0.6707	0.8834	0.8504	0.9096
1.0	0.7556	0.7422	0.6701	0.8829	0.8559	0.9048

The results in Table 3 demonstrate that with value of *β* ranging from 0.1 to 0.9 overall, the AUC values of combined path with length 2, 3, 4 gradually increase. However, its AUC value is slightly decreased from 0.9 to 1.0. Therefore, HSDD performs best when *β* is at 0.9 and combined path is with length 2, 3, 4. For other path combinations, the best value for *β* can also be obtained from Table 3.

At the same time, we also evaluate the effect of path combination on HSDD. Results in Table 3 show that combined paths performs better than independent paths. Combined path with length 2, 3, 4 achieves the best performance comparing with other path combination. This is because the combined path with length 2, 3, 4 has more significant meanings than combined path with length 2, 3 and combined path with length 3, 4. Therefore, we can set *β* at 0.9 and select combined path with length 2, 3, 4 as the best path combination for HSDD, which can most effectively measure the associations between drugs and diseases. The phenomenon of AUC variations with path combination is consistent with previous research on path-based algorithms [51].

### Case studies

To verify the effectiveness of HSDD further, we will utilize HSDD infer novel drug-disease associations. In this experiment, we select five common diseases [12] and then predict their related drugs using HSDD. These five disease are Huntington disease (HD, OMIM 143100), Non–small-cell lung cancer (NSCLC, OMIM 211980), Alcohol dependence (AD, OMIM 103780), Small-cell lung cancer (SCLC, OMIM 182280) and Polysubstance abuse, Susceptibility to (PSAB, OMIM 606581). For each disease, we firstly obtain the known drugs and then present the top ten predicted drugs, which has shown in Table 4. We take Huntington disease as an example to explain the results of case study.

**Table 4:** Case study results: the top ten predicted drugs for selected diseases

Disease Name	Known drugs (DrugBank IDs)	Top 10 ranked predictions
Huntington OMIM ID: 143100	Baclofen (DB00181) Tetrabenazine (DB04844)	Quetiapine (DB01224), Olanzapine (DB00334), Bupropion (DB01156), Clozapine (DB00363), Carbidopa(DB00190), Metyrosine(DB00765), Phentermine (DB00191), Pethidine(DB00454), Phenelzine(DB00780) Donepezil (DB00843)
NSCLC OMIM ID:211980	Doxorubicin (DB00997)	Daunorubicin (DB00694), Idarubicin (DB01177), Valrubicin (DB00385), Oxymorphone (DB01192), Anastrozole (DB01217), Oxycodone (DB00497), Buprenorphine (DB00921), Levobunolol (DB01210), Vincristine (DB00541), Carboplatin (DB00958)
AD (OMIM ID: 104300)	Citalopram (DB00215), Chlordiazepoxide (DB00475), Acamprosate (DB00659), Naltrexone (DB00704), Disulfiram (DB00822), Ondansetron (DB00904)	Galantamine (DB00674), Olanzapine (DB00334), Risperidone(DB00734), Escitalopram (DB01175), Terfenadine (DB00342), Alprazolam (DB00404) Diazepam (DB00829), Lorazepam (DB00186), Methimazole (DB00763), Mechlorethamine (DB00888)
SCLC (OMIM ID: 182280)	Cisplatin (DB00515) Methotrexate (DB00563) Teniposide (DB00444) Etoposide (DB00773) Topotecan (DB01030)	Lithium (DB01356), Mechlorethamine (DB00888), Carboplatin (DB00958), Epirubicin (DB00445), Daunorubicin (DB00694), Doxorubicin (DB00997), Irinotecan (DB00762), Codeine (DB00318), Vinorelbine (DB00361), Frovatriptan (DB00998)
PSAB, (OMIM ID: 606581)	None	Citalopram (DB00215), Chlordiazepoxide (DB00475), Acamprosate (DB00659), Naltrexone (DB00704), Disulfiram (DB00822), Ondansetron (DB00904), Niacin (DB00627), Clofibrate (DB00636), Fenofibrate (DB01039), Gemfibrozil (DB01241)

Huntington’s disease (HD), also known as Huntington’s chorea, is an autosomal-dominant, progressive neurodegenerative disorder with a distinct phenotype and can results in death of brain cells [52, 53]. In OMIM database, HD has many phenotypes and here we select 141,300 as its phenotype to predict its related drugs.

HSDD has predicted ten drugs for HD. Quetiapine (DB01224) was studied in five consecutive patients with Huntington’s disease in a long-term facility. These patients behave improvement of behavioral symptoms without worsening of motor functioning [54]. Author Paleacu designed an experiment of eleven HD patients and the results clearly demonstrates that Olanzapine (DB00334) is safe and is an effective treatment for the behavioral disturbances and frequently for the chorea seen in HD patients [55]. Besides, to evaluate the efficacy and safety of Bupropion (DB01156) in the treatment of apathy in Huntington’s disease (HD), Gelderblom conducted a multicenter, randomized, double-blind, placebo-controlled, prospective crossover trial [56]. The results of the trail show that bupropion does not alleviate apathy in HD. However, the author observed the effects of participation/placebo, which document the need for carefully controlled trials. For other diseases, the predicted drugs have been presented in Table 4. In this experiment, when measuring HeteSim scores of drug and disease pairs, we utilize all the information in the network including all the known drugs. Most of predicted drugs predicted by HSDD are supported by literature, which indicates its good performance.

## Discussion

In this study, we proposed HSDD to infer the associations between drugs and diseases.

Comparing with other effective methods, HSDD shows best performance in all datasets. HSDD has the ability to capture the sematic meaning of meta-paths in the heterogeneous network. Besides, the experimental results show that HDSS performs best with the combined path length 2, 3 and 4. This is because this conbined path can extracting much more meaningful meta-path from the drug-disease heterogeneous network than the other paths. In the end, the results of HSDD on case studies indicate its good performance, which is validated by literature.

## Conclusions

Drug repositioning is a promising and efficient way to develop the associations of drugs and diseases. With the rise of precision medicine, drug repositioning will play a more and more important role. In this study, we proposed a novel method called HSDD to research drug repositioning problem. HSDD makes the best use of meta-paths with different lengths in the drug-disease heterogeneous and measures their association strength based on HeteSim scores. The results in all the cross validation experiments show that HSDD outperforms other methods, which can effectively improve the prediction performance. Besides, case studies for some typical diseases indicate that HSDD is an efficient useful way to predict potential drug-disease associations.

HSDD can be extended easily to other research as long as the data is available and suitable. For example, RNA-protein association prediction is another meaningful study. Similar to drug repositioning, network-based methods have already achieved a good performance. Further, the identification of microRNAs associated with diseases is very important for understanding the pathogenesis of diseases at the molecular level. HSDD can be widely used in these applications.

At the same time, we plan to address two issues in future work. First, we only consider the paths with length less than five in this study. As we know, longer paths also have significant meanings. Therefore, we should investigate the effect of other longer paths on HSDD more comprehensive. Secondly, in this study we only consider the direct associations of drugs and diseases, which only utilizes two kinds of objects. Some research has put drug-target relationships into drug repositioning. For example, we can predict drug disease associations based on a drug-target-disease three-layer- heterogeneous network, which is inspired by data fusion.

## Abbreviations

DBSI: Drug-based similarity inference
GBA: Guilt-by-association (GBA)
HSDD: HeteSim_DrugDisease
NBI: Network-based inference
TBSI: Target-based similarity inference
